# Tumor size classification of the 8^th^ edition of TNM staging system is superior to that of the 7^th^ edition in predicting the survival outcome of pancreatic cancer patients after radical resection and adjuvant chemotherapy

**DOI:** 10.1038/s41598-018-28193-4

**Published:** 2018-07-10

**Authors:** Lin Cong, Qiaofei Liu, Ronghua Zhang, Ming Cui, Xiang Zhang, Xiang Gao, Junchao Guo, Menghua Dai, Taiping Zhang, Quan Liao, Yupei Zhao

**Affiliations:** Department of General Surgery, Peking Union Medical College Hospital, Chinese Academy of Medical Sciences & Peking Union Medical College, 100730 Beijing, China

## Abstract

The 8^th^ edition of TNM staging system has been released and it incorporates many changes to the T and N classifications for pancreatic cancer. Comparative study between the 7^th^ and 8^th^ edition of TNM staging system from Asian population has not been reported yet. This study aimed to compare the 7^th^ and 8^th^ edition of staging system for pancreatic cancer by using a cohort of pancreatic cancer patients from China after R0 pancreaticoduodenectomy and adjuvant chemotherapy. The results showed according to the pT classification of 7^th^ edition, pT3 was predominant (87.25%), however, the new edition led to a more equal distribution of pT classification. pT1, pT2 and pT3 was 27.45%, 56.86% and 15.69%, respectively. According to the new pN classification, 18.63% of the patients were pN2. The pT classification in the 8^th^ edition was significantly superior to that in the 7^th^ edition at stratifying patients by overall survival. The pN classification in the 8^th^ edition failed to show an advantage over the 7^th^ edition in stratifying patients by overall survival. Therefore, the new pT classification, but not the new pN classification, showed a significant advantage over the previous edition at predicting the overall survival of pancreatic cancer patients.

## Introduction

Despite tremendous efforts to elucidate the mechanisms underlying the initiation, progression and metastasis of pancreatic ductal adenocarcinoma (PDAC), its 5-year overall survival remains approximately 8.2% in America^1–3^. During the last several decades, the incidence of PDAC has increased in both Western countries and China^4,5^.

Accurate evaluation of tumor stage is a prerequisite for further treatment and prognostic prediction. The AJCC/UICC TNM staging system has been widely applied worldwide as the most authorized tool for tumor staging assessment. The first edition was released in 1977, and it has been updated several times every 5–7 years. The 5^th^ edition for pancreatic cancer was released in 1997, and no changes have been made in the 6^th^ and 7^th^ editions in the last 20 years^6^.

In October of 2016, AJCC/UICC released the 8^th^ edition, which incorporated significant changes in the T and N classification of PDAC. In the 8^th^ edition, stages T1-T3 are redefined according to tumor size (T1 ≤ 2 cm; 2 cm ≥ T2 ≤ 4 cm; T3 > 4 cm). When the tumor invades the celiac axis, common hepatic artery and/or superior mesenteric artery, it is defined as T4, and the classification as “unresectable” was removed. In the 8^th^ edition, the N classification was further subdivided according to the number of positive lymph nodes as N0, N1 (≥1 and ≤3) and N2 (>3). In the 8^th^ edition, T1–3N2M0 was defined as stage III, and the other stages remained unchanged (Table 1).

**Table 1:** The definitions of the 7^th^ and 8^th^ edition of TNM staging system of pancreatic cancer by AJCC/UICC.

	7^th^	8^th^		7^th^			8^th^		
T1	Tumor limited to the pancreas, ≤2 cm in greatest dimension	Maximum tumor diameter ≤2 cm		**T**	**N**	**M**	**T**	**N**	**M**
T2	Tumor limited to the pancreas, >2 cm in greatest dimension	Maximum tumor diameter >2, ≤4 cm	**IA**	T1	N0	MO	T1	N0	M0
T3	Tumor extends beyond the pancreas but without involvement of the celiac axis or the superior mesenteric artery	Maximum tumor diameter >4 cm	**IB**	T2	N0	M0	T2	N0	M0
T4	Tumor involves the celiac axis or the superior mesenteric artery (unresectable primary tumor)	Tumor involves the celiac axis, common hepatic artery or the superior mesenteric artery	**IIA**	T3	N0	M0	T3	N0	M0
N0	No regional lymph node metastasis	No regional lymph node metastasis	**IIB**	T1-T3	N1	M0	T1-T3	N1	M0
N1	Regional lymph node metastasis	Metastasis in 1–3 regional lymph nodes	**III**	T4	any N	M0	T4 (any T)	any N (N2)	M0
N2	—	Metastasis in ≥ 4 regional lymph nodes	**IV**	any T	any N	M1	any T	Any N	M1
M0	No distant metastasis	No distant metastasis							
M1	Distant metastasis	Distant metastasis							

Recently, the superiority of the 8^th^ edition at stratifying patients by survival was evaluated in two validation studies from America and two studies from Germany; however, the results were inconsistent. At present, the superiority of the 8^th^ edition to the 7^th^ edition at predicting the prognosis of PDAC has not been evaluated in an Asia population^7–10^. Since many confounding factors, such as tumor location, tumor margin^11–13^, and adjuvant chemotherapy^14^, could affect the clinical value of the TNM staging system in predicting patients survival, we rigorously enrolled 102 PDAC patients who underwent R0 pancreaticoduodenectomy and at least three cycles of gemcitabine-based chemotherapy with a long follow-up period to validate the potential superiority of the new TNM staging system in stratifying patients based on survival.

## Results

### Patient demographics

One hundred two patients were enrolled, and survival information was available for all patients. The follow-up time ranged from 36 to 90 months. The median survival was 32.00±4.91 months (95% CI 22.37±41.62), and the 1-, 2- and 3-year survival rates were 88.2%, 70.5%, and 40.5%, respectively. The detailed clinicopathological information was provided in Table 2.

**Table 2:** Patient demographics.

Characteristics	N0. of patients
Age	
≤60 y	51
>60 y	51
Gender	
Male	66
Female	36
pT classification (8^th^)	
pT1 (≤2 cm)	27
pT2 (>2 cm, ≤4 cm)	57
pT3 (>4 cm)	18
pT classification (7^th^)	
pT1	7
pT2	6
pT3	89
pN classification (8^th^)	
pN0 (0)	40
pN1 (1~3)	43
pN2 (≥4)	19
TNM stage (7^th^)	
IA	5
IB	2
IIA	34
IIB	61
TNM stage (8^th^)	
IA	12
IB	23
IIA	6
IIB	44
III	17
Differentiation	
Well/moderate	75
Poor	27
Perineural invasion (PNI)	
Yes	21
No	81
Micro-cancerous embolus	
Yes	12
No	90
CA19-9	
≤34U/ml	27
>34U/ml	74
NA	1
CA242	
≤20U/ml	46
>20Uml	41
NA	15
CEA	
≤5 μg/ml	79
>5 μg/ml	21
NA	2
Perioperative bile drainage	
Yes	45
NO	57
Diabetes mellitus	
Yes	20
No	82

NA: Not Available.

### Comparison of the 7^th^ and 8^th^ editions of the TNM staging system for patients

Stages pT1 and pT2 in the 7^th^ edition were well matched with those in the 8^th^ edition, but only 18/89 (20.2%) stage pT3 cases in the 7^th^ edition were matched with those in the 8^th^ edition (Table 3) 60.78% of patients had lymph node metastasis, and according to the new pN classification, 18.63% of these patients had metastasis in more than 3 lymph nodes (pN2) (Table 4). Stages IA and IB in these two editions were well matched. According to the 7^th^ edition, 33.3% and 59.8% of patients were stage IIA (T3N0M0) and IIB (T1–3N1M0), respectively. In the new edition, only 5.9% of the patients were stage IIA (T3N0M0); 22.5%, 43.1%, and 16.7% of the patients were stage IB (T2N0M0), IIB (T1–3N1M0) and III (T1–3N2M0), respectively. Moreover, 6/34 (17.7%) stage IIA cases in the 7^th^ edition were matched with those in the 8^th^ edition, and the others were characterized as stage IA and IB by the 8^th^ edition. A total of 17/61 (27.9%) stage IIB cases in the 7^th^ edition were considered stage III by the 8^th^ edition (Table 5).

**Table 3:** The comparison of pT classifications of 7^th^ and 8^th^ edition.

8th	pT1	pT2	pT3	Total
7th				
pT1	7			7
pT2		6		6
pT3	21	50	18	89
Total	28	56	18	102

**Table 4:** The comparison of pN classifications of 7^th^ and 8^th^ edition.

8^th^	pN0	pN1	pN2	Total
7th				
pN0	40			40
pN1		43	19	62
Total	40	43	19	102

**Table 5:** The comparison of TNM staging system of 7^th^ and 8^th^ edition.

8^th^	IA	IB	IIA	IIB	III	Total
7th						
IA	5					5
IB		2				2
IIA	7	21	6			34
IIB				44	17	61
Total	12	23	6	44	17	102

### The ability of the 7^th^ and 8^th^ editions of the TNM staging system to stratify patients by overall survival

**Figure 1:**
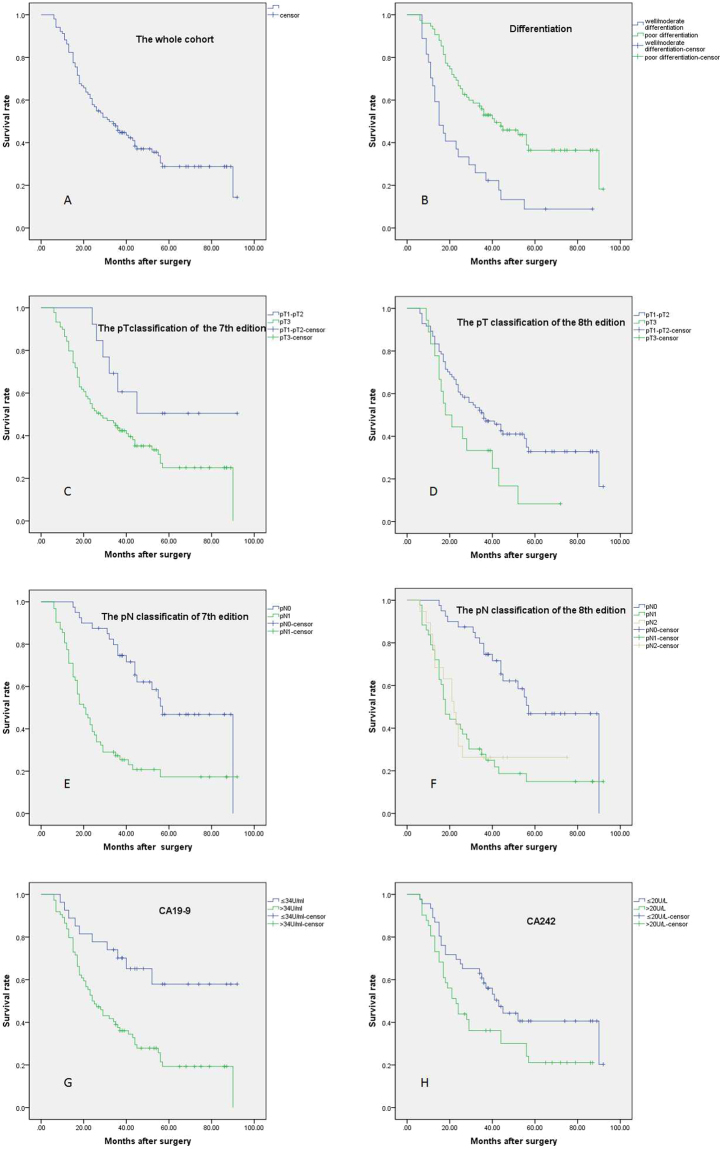
Survival curves of patients with different clinicopathological characteristics. (**A**) Overall survival of all patients. (**B**) Poor differentiation predicted worse prognosis (*P* < 0.0001). (**C**) The pT classification in the 7^th^ edition failed to stratify by prognosis (*P* = 0.054). (**D**) The pT classification in the 8^th^ edition successfully stratified by prognosis (*P* = 0.033). (**E**) The pN classification in the 7^th^ edition stratified by prognosis (*P* < 0.0001). (**F**) The survival of patients classified as pN1 and pN2 in the 8^th^ edition was not significantly different (*P* > 0.05). (**G**) The OS was worse for patients with elevated CA19-9 than for those with decreased or normal CA19-9 (*P* = 0.001). (**H**) The OS of patients with elevated CA242 was worse than that of the other patients (*P* = 0.044).

**Figure 2:**
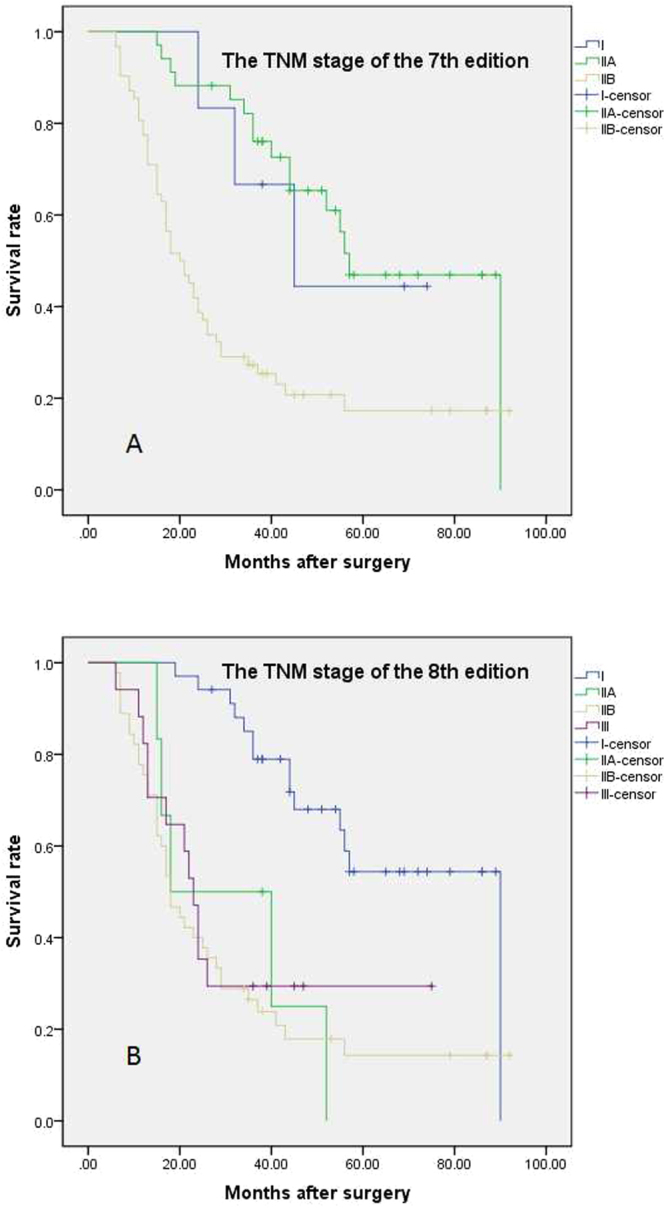
Survival curves based on TNM staging systems. (**A**) The 7^th^ edition TNM staging system failed to discriminate patients with stage I disease from those at other stages by overall survival. (**B**) The 8^th^ edition TNM staging system could differentiate patients with stage I disease from those at other stages by overall survival.

After multivariate analysis, CA19-9, tumor size larger than 4 cm (pT3 of the 8^th^ edition), poor differentiation and positive lymph node metastasis were independent risk factors for poor survival, but pT classification in the 7^th^ edition and the number of positive lymph nodes (pN1 and pN2 classification in the 8^th^ edition) failed to stratify patients by survival; this finding indicated that pT classification in the 8^th^ edition was superior to that in the 7^th^ edition. However, pN classification in the 8^th^ edition did not show significant superiority to that in the 7^th^ edition at stratifying patients by overall survival (Table 6, Fig. 1). In addition, the 8^th^ edition of the TNM staging system successfully stratified stage I patients from those at other stages based on overall survival, which the 7^th^ edition could not do (Fig. 2).

**Table 6:** Univariate analysis and multivariate analysis of the overall survival of the patients.

Variable	NO. of patients	Univariate analysis			Multivariate analysis		
		Median ± SE	*95% CI*	*P* value	*HR*	*95% CI*	*P* value
Age				0.092			
≤60 y	51	25.00 ± 4.59	16.00–34.00				
**>**60 y	51	43.00 ± 7.75	27.81–58.20				
Gender				0.677			
Male	66	31.00 ± 6.14	18.97–43.03				
Female	36	34.00 ± 8.74	16.86–51.14				
T stage (8^th^)				0.033*	1.69	0.91–3.13	0.049^*^
≤4 cm (pT1~T2)	84	36.00 ± 6.20	23.84–48.16				
**>**4 cm (pT3)	18	18.00 ± 4.24	9.68–26.32				
T stage (7^th^)				0.056			
pT1~T2	13	38.00 ± 8.40	21.70–49.20				
pT3	89	27.65 ± 6.40	16.00–42.60				
Lymph node metastasis				<0.0001^**^	3.29	1.75–6.20	<0.0001^**^
pN0	40	57.00 ± 5.89	45.47–68.53				
pN1	62	20.00 ± 2.61	14.86–25.14				
Differentiation				<0.0001^**^	0.47	0.26–0.82	0.008^**^
Well/moderate	75	41.00 ± 8.32	24.69–57.31				
Poor	27	15.00 ± 2.60	9.91–20.09				
Micro-cancerous embolus				0.571			
Yes	12	26.00 ± 7.75	10.81–41.19				
No	90	34.00 ± 6.37	21.51–46.49				
Perineural invasion (PNI)			0.540				
Yes	21	24.00 ± 6.87	10.54–37.46				
No	89	36.00 ± 5.47	25.27–46.73				
CA199				0.001^**^	2.74	1.12–6.67	0.027^*^
≤34U/ml	27	NA	NA				
**>**34U/ml	74	24.00 ± 3.31	17.51–30.49				
CA242				0.044	1.09	0.61–1.97	0.744
≤20U/ml	46	43.00 ± 5.28	32.65–53.35				
**>**20Uml	41	23.00 ± 3.20	16.73–29.27				
CEA				0.861			
≤5 μg/ml	79	35.00 ± 5.04	25.12–44.88				
**>**5 μg/ml	21	24.00 ± 16.96	0–47.25				
Perioperative bile drainage				0.151			
Yes	45	26.00 ± 6.02	14.19–37.81				
No	57	40.00 ± 8.33	23.68–56.33				
Diabetes mellitus				0.133			
Yes	20	55.00 ± 13.62	28.30 ± 81.7				
NO	82	28.00 ± 4.03	20.11–35.89				

*P < 0.05; **P < 0.01.

## Discussion

Since the 1^st^ edition of the TNM staging system was published in 1977, the AJCC/UICC has released a total of eight editions. During the last 40 years, considerable improvements have been achieved in many malignancies, such as melanoma, gastric cancer, colorectal cancer, breast cancer and lung cancer. However, despite tremendous efforts, a diagnosis of PDAC remains devastating^15–18^. There has not been any change in the TNM staging system for PDAC in the last three editions. Finally, in the new 8^th^ edition, many changes in both T and N classification for PDAC have been incorporated, but whether these changes make the 8^th^ edition superior to the 7^th^ edition at stratifying patients by prognosis remains unclear.

The role of the previous T classification in predicting the prognosis of PDAC patients is controversial^19^. It is difficult to accurately ascertain the T classification according to the previous editions; in the 8^th^ edition, only tumor size was considered, significantly increased the accuracy of the pathological assessment^7,10^. The number of positive lymph nodes was adopted to define the N classification in the new edition, but the role of this factor remained unclear^20–22^. Allen *et al*.^7^ analyzed 2318 cases of PDAC after resection from 3 large volume centers in America and found that the 8^th^ edition increased the reproducibility of the T3 classification among different centers and that the N classification in the 8^th^ edition was able to discriminate the prognosis of patient subgroups. Kamarajah *et al*.^10^ collected data from the Surveillance, Epidemiology and End Results (SEER) database from 2004 to 2013 and analyzed 8960 pancreatic cancer patients without metastasis who underwent surgical resection. The results showed that the 8^th^ edition allowed for finer stratification of patients according to the extent of nodal involvement. Although these two studies had a large number of patients, there were some limitations: (1) all the data were from America; (2) the time interval during which patients were enrolled was more than 10 years; and (3) information on adjuvant treatment was missing. More recently, the results of two validation studies from Germany were inconsistent with the significance of the N classification in the 8^th^ edition. Welsch *et al*.^9^ reported a cohort of 256 PDAC patients who underwent curative resection from 2005 to 2015, and the results showed that the new N and T classifications both better discriminated PDAC patients by survival. Schlitter *et al*.^8^ reported two cohorts of 523 PDAC patients from Germany who underwent surgery in two hospitals over two decades (1991–2006; 2007–2014). They found that the T classification in the 7^th^ edition could not discriminate patient prognosis, whereas the 8^th^ edition showed substantial success in stratifying patients by prognosis. The new N classification failed to show high clinical relevance in either cohort.

Herein, we report the first validation of the 8^th^ edition in an Asia population. To test the reliability of stratification based on the new TNM staging system, we enrolled patients according to rigorous criteria to avoid confounding factors to the maximal extent possible: (1) only patients treated after 2010 were enrolled; (2) there was a long follow-up period; (3) all cases achieved R0 resection; (4) only patients with tumors located in the head of the pancreas were enrolled; and (5) all the enrolled patient underwent at least 3 cycles of adjuvant chemotherapy based on gemcitabine. The median age of the patients was 60 years, which was a little younger than that of the above reports from Germany and America. There were slightly more male patients than female patients (1.8:1.0), which was in accordance with the ratio reported in the above studies from Germany. CA19-9, CA242 and CEA were elevated in 72.5%, 40.5% and 20.6% of the patients, respectively. Some previous studies have reported that elevated CA19-9 and CA242 are risk factors for poor prognosis^23,24^; in this study, we also found that elevated CA19-9 and CA242 were associated with poor prognosis.

Overall, 60.7% of the patients had lymph node metastasis, and 42.2% and 18.5% of the patients were pN1 and pN2, respectively. The pN classification in the 7^th^ edition successfully stratified patient based on survival, but the new pN classification did not show an advantage over the 7^th^ edition in discriminating patients by prognosis. In the 7^th^ edition, 87.3% of patients were pT3, whereas 54.9% of patients were pT2 in the 8^th^ edition, which was similar to the percentage reported in the above studies from America and Germany. pT3 classification in the 7^th^ edition was redefined as pT1, pT2 and pT3 classification in the 8^th^ edition, with a predominance of pT2 (51.2%); only 20.2% of pT3 classifications in the 8^th^ edition corresponded with those in the 7^th^ edition. pT classification in the 7^th^ edition failed to stratify patients by survival. However, the prognosis of patients classified by the 8^th^ edition as pT1-pT2 or pT3 was significantly different, suggesting that the new T classification was superior to the previous one at stratifying patients by survival. Although the 8^th^ edition of the TNM staging system did not stratify patients by survival for all stages, it could discriminate stage I patients from those at other stages by survival, which the 7^th^ edition of the staging system failed to do, further showing the superiority of the new edition.

In conclusion, this study is the first to validate the 8^th^ edition of the TNM staging system for PDAC in an Asian population after its release. Compared to the validation studies from America and Germany, this study was performed at only a single center with a relatively small number of patients, which may weaken the reliability of the results. As previously stated, to validate the value of the TNM staging system, we enrolled patients according to rigorous criteria to minimize possible confounding factors that may affect the efficacy of the TNM staging system. The results supported that the new pT classification had a substantial advantage over the previous edition in predicting the overall survival of PDAC patients undergoing R0 pancreaticoduodenectomy and gemcitabine-based adjuvant chemotherapy. In this study, the new pN classification failed to show superiority over the previous edition in stratifying patients by overall survival. To further validate the superiority of the new edition of the TNM staging system at stratifying PDAC patients in Asia (China) by survival, a multicenter study with a large number of patients will be needed.

## Methods

### Patients and follow-up

In total, 102 consecutive cases of PDAC were enrolled from January 2010 to October 2014 according to the following inclusion criteria: (1) the final pathological examination confirmed PDAC; (2) none of the patients underwent neoadjuvant treatment; (3) radical R0 pancreaticoduodenectomy was achieved (microscopic margin > 1 mm); (4) all the patients underwent at least three cycles of gemcitabine-based adjuvant chemotherapy; (5) information on postoperative survival time was available, and patients who died within 3 months after surgery were excluded; and (6) all the patients signed the informed consent form. The clinicopathological information, including age, gender, tumor size, differentiation, perineural invasion (PNI), micro-cancerous embolus, CA19-9, CA242, CEA, pT classification, pN classification and TNM stage of the 7^th^ and 8^th^ editions, was extracted from the PACS system. After operation, the patients were followed up every 3∼6 months by outpatient clinic visits or telephone calls until patient death. Overall survival (OS) was defined as the survival time after surgery. All the patients agreed to donate bio-specimens for scientific research and publication and signed the informed consent form before surgery. The study was approved by the ethics committee of Peking Union Medical College Hospital.

### Statistics

IBM SPSS Statistics software version 22.0 was applied for statistical analysis. Overall survival was analyzed using the Kaplan-Meier method, and the values were compared using the log-rank test. Multivariate analysis was performed using the Cox proportional hazard model. A P value less than 0.05 indicated statistical significance.

### Data Availability

The primary data is available if reasonable requested.

### Ethical approval

All procedures involving human participants were in accordance with the ethical standards of the ethical committee of Peking Union Medical College Hospital and with the 1964 Helsinki declaration and its later amendments or comparable ethical standards. All of the patients signed the informed consent for the scientific use of their information or bio-specimen before surgery.
